# An Account of the Constitution and Management of the Cotton-Mills in Lower Austria. With Especial Reference to the Moral, Intellectual, and Physical Education of the Children Employed in Them, and to the Legal Regulations Subsisting in This Respect

**Published:** 1844-07

**Authors:** 


### Art. XII.

*Darstelluug der Verfassung und Einrichtung der Baumwoll-Spinnerei-Fabriken in Nieder-Oesterreich. Mit besonderer Beziehung auf die moralisch-intellectuelle und physiche Erziehung der daselbst verwendeten Kinder und die diessfalls bestehenden gesetzlichen Vorschriften.* Von Dr. Jos. Joh. Knolz.—*Wien*, 1843.

*An Account of the Constitution and Management of the Cotton-mills in Lower Austria. With especial reference to the moral, intellectual, and physical education of the children employed in them, and to the legal regulations subsisting in this respect.* By J. J. Knolz, m.d.—*Vienna*, 1843. 8vo, pp. 96.

A brief period only has elapsed since we had occasion to examine, at some length, the oft-discussed question regarding the sanitary influence of the factory system ; and our investigations brought us to conclusions varying, to some extent, from those ordinarily prevailing with respect to this matter. It did not appear to us, upon any well-founded evidence, that cotton-mills, in their prejudicial influence upon the public health, differed materially from other work-places which furnish employment to large numbers of the population. That health was deteriorated, and life shortened, by factory working, particularly in childhood, we never doubted ; we considered, however, that a similar result flowed, more or less, from almost every occupation characterizing and associated with a high state of civilization ; and again, that, whatever ills did really arise to health from the *industrial* relations of large numbers of artisans, their *domestic* condition was yet more in fault. It was clear to us that the position of the labouring classes, as a whole, was far less compatible with health and longevity than that of the superior grades ; that much that was injurious in these regards resulted, however, from an aggregation of the evils brought to light by recent researches into the state of our modern cities ; and, in a word, that in the instance of our manufacturing population, the sanitary grievances came much more from the *great town* than the *factory system.*

It was not our object to constitute ourselves the apologists of the factory system, and still less to deny the deleterious consequences resulting from the wholesale employment of young children in cotton-mills, as at present constituted : our aim was to strip this question of much of its garniture that was false and exaggerated, in order that the actual evil might be recognized at its true source ; and it seemed to us that this was but little likely to occur whilst violent and unjust denunciations of factories and their occupiers, took place on the one hand, to be neutralized by excessive and partisan panegyric on the other. We had not, nor have we, any notions or views upon this subject, other than those which have issued from an impartial examination of the evidence ; and, in committing to paper the conclusions at which we may have arrived, we are influenced by no considerations foreign to our professed purposed, that of anxiously striving, with truth for our cynosure, to attain accuracy in our statements, and justice in our criticism ; a principle, we may add, which can alone guide the progress of this Journal under its present management.

We have been led to these remarks, in brief recapitulation of our former argument, by the perusal of the concise and valuable little work, the title of which we have given above ; a brochure published in the course of last year, and full of interest from the circumstance of its demonstrating by its statements the correctness of our position, that factory working *per se* is not necessarily more prejudicial to health than most other occupations, but that the ills which in many cases have arisen from and been associated with this branch of industry are of a nature, to a considerable extent at least, admitting of removal. We think it most important that just notions regarding this question should obtain currency, for it is one which has been, and is likely yet to continue, a subject of legislation with a view to improvement; and, under present circumstances, we very much think that any mere shortening of the hours of labour, hitherto the principally prescribed method of amelioration, would only diminish to the workpeople the means of supplying themselves with due sustenance, and at the same time condemn them for a longer period to breathe the impure atmosphere of their own dwellings, and in such way operate still more deterioratingly upon their health.

We gather from Dr. Knolz's publication that, in Lower Austria, the hours of labour, in the cotton-mills, are more extended than in our own country, and yet that, generally speaking, the health of the operatives is in a better condition than that of other classes of workpeople,—a result attributable not, we conceive, to the character of the occupation itself, but to the excellent regulations, hygienic and otherwise, employed by the authorities in the management of this department of administration. As involving points of considerable interest in themselves, and as indicating, in our judgment, the direction in which legislative interference should run, we shall supply to the reader some of the particulars ; and although the special circumstances of different countries will but rarely allow one state to take another as an exact pattern, even in things relating to the subject of our present discussion, yet the exhibition by one nation of a broad truth, developed by experience, may always be rendered, in some measure, available by every other, bent on progressive advance and improvement. We proceed to furnish a brief analysis of some of the matters contained in the little work now before us.

The factories described by our author are situated, for the most part, not in the centre of large cities, but in hamlets, villages, and small country towns; localities, generally speaking, through which great brooks take their course, the waters of which can be turned to account in the prosecution of the work. Most of these districts are open plains at the extremities of the villages, well exposed to the air, and so far most advantageously situated with regard to hygienic conditions. All considerations relating to security against fire, and the healthfulness of the foundations, and other collateral circumstances of this kind, are determined by the authorities, prior to the erection of the mills ; and it is stated that, even if no legal regulations subsisted in this respect, the disposition of the capitalist would generally direct him to entertain, in his projects, the question regarding the probable health of his future workpeople. In building the required structures, every care is bestowed upon the height and the width of the rooms, and upon the facilities for ventilating them ; and, further, in the immediate vicinity of the workplaces, are situated dwellings of a very superior character, the property of the mill-owner, for the operatives themselves, as well as apartments for their treatment in sickness; and, when schools do not otherwise exist in the place, certain divisions of the erection are appropriated for this purpose, and also as residences for the teachers. The due management of all these matters is rendered obligatory upon the occupiers of the establishment. Under all these circumstances, it is affirmed that the children of the operatives, many of whom are associated with their parents in factory-working, are better educated and fed than those of the other villagers, and, being removed from the bad example furnished by artisans in large towns, are strangers to the vices and delinquencies attributed to corresponding classes in France and England.

With respect to the age of the children employed in the cotton-mills, it is at the present time seldom that any are admitted under the age of twelve, the interest of the occupier, in the estimation of our author, demanding that younger children should not receive admittance, as from frivolity, want of thought, and general unfitness, they would too often be likely to occasion mischief. However, occasionally, they are received into the mills so early as the age of nine, from consideration to the parents, who, being themselves employed in the factories, could not always, if the contrary rule admitted of no exception, retain a suitable watch over their moral and physical welfare; and the children out of work might thus pursue, uncontrolled, the road to destruction. These infant labourers, however, are stated to be very few; and in all the mills of Lower Austria, those from nine to twelve years old do not amount to a twentieth of the children; and these latter come to about one fourth of the whole number of the employed.

The time of labour for those of twelve years of age, is about six hours, or six hours and a half, in the forenoon, and the same period in the afternoon, with an interruption of an hour and a half at mid-day.

The species of occupation exacted from children in these estsblishments requires but little bodily exertion; and, as a matter of prudence in the management of the business, the division of labour is governed most carefully by the physical strength and capability of those to whom particular kinds are awarded. It consists, among the elder children, in the placing of cotton upon the carding-machines, in the carrying to and fro of small tin cases filled with cotton-wool, and such like light employment; and, with the younger, in piercing the threads and screwing the caps, at the spinning-machines. The continuance of this exceedingly easy and simple work is rendered still less fatiguing by the fact of its requiring a frequent change, both of posture and place. Labour requiring any exertion is consigned altogether to adult persons. The author bestows a glowing eulogy upon the mildness, the probity, and the general excellence of the young people engaged in these establishments, and the result he assigns to the wise and benevolent discipline to which they are systematically subjected. He affirms that nowhere, in his part of the world, are children treated with the barbarity which has obtained in some districts of England; that such a disposition does not exist in the character of the mill-owners, and that any deviation in this point of view would quickly be visited, not only with the indignation of the people, but with an interference of the government. From these last remarks, and some others occurring in the course of the work, we are led to believe that the author's notions respecting our own manufactures have been obtained, directly or indirectly, from the publication of the parliamentary evidence on which we animadverted at some length in our former article. In referring to the way in which the operative classes are treated in the Austrian factories, he observes that, beyond the care arising from the paternal character of the government regulations, and the disposition of the masters, they are necessitated by a regard to their own interests, in consequence of the great demand for labour, to protect and welltreat the workpeople, as well as to provide for excellence in the sanitary state alike of the mills, dwellings, sick-wards, and the schools.

We have given the above description of the physical conditions in which the Austrian factory-people are placed, as a just illustration of a reasonable and practicable state of things, calculated to exhibit, in an isolated manner, the destructive effects to health, if any, resulting from the working in factories; inasmuch as here most of the other provocatives to disease, commonly obtaining amongst corresponding classes in this country, seem to be withdrawn.

Now, if the amassing of individuals in mills, to the extent necessary to the successful prosecution of the work, and the required temperature, and the demanded constraint of position, and so on, were really the cause of all the horrors, revealed or anticipated, by many who have dwelt upon this subject, we should certainly have them displayed even in Austria; for it seems that the establishments are usually in full vigour of operation for somewhere about fifteen hours a day, and the young children are engaged in actual work for about thirteen hours. Let us, then, see what Dr. Knolz states upon this matter—the account which he has furnished being stamped, so far as it can be judged from style, with all the outward characteristics of candour and truth.

Our author very properly premises that the physical condition of the children employed in the factories may be deduced from the circumstances in which they are placed, as already described. They work, he says, everywhere, in the mills in question, in lofty rooms, which, on account of the business itself, are kept in the cleanest state possible, being in summer most carefully ventilated, and in winter well heated by means of Messner's stoves, or by a circulation of hot water in tubes. Their occupation, he repeats, is light, requiring varying postures; and, in every way, they are better protected against influences prejudicial to health than young people engaged in other kinds of work. He goes on to say, that their excellent state of health must strike every observer who sees them, at the close of day, leave the workplaces in a frame of mind the most cheerful conceivable. The larger mills have been in operation now for almost thirty-six years, within which time there must have been a change in the children employed at least nine times, yielding, within one generation, according to a calculation made, an aggregate of 21,000 individuals who passed four of the best years of their childhood in factory labour; a circumstance, he affirms, proving by a long experience, that no needful damage is done to young children by this occupation, considered in and for itself, and with an exclusion of all the other removable sources of physical deterioration. Notwithstanding so large a proportion of the villagers have, for so many years, been as it were brought up in the mills, not the slightest trace of a crippled or stunted race is discoverable in the vicinity, either during a military conscription, in the medical visitations periodically taking place, or in the hospitals or alms-houses. In the ravages of recent epidemics, which always devastate and scatter away the feebler members of any community, experience furnishes a still more important demonstration, how carefully the health of the factory-people is preserved. Everywhere, near the larger mills, infirmaries are instituted, which are furnished with well-paid physicians, surgeons, in order that all—adults as well as children—may receive appropriate care and attention during illness; and, in all cases of epidemic disease, the mill-owner is compelled by existing regulations to provide everything at his own cost. As the successful result of all these arrangements, it is stated by Dr. Knolz that the instance may be adduced of what obtained during the prevalence of cholera in 1832, in the largest factory in the monarchy, at Pottendorf, where upwards of 1200 persons are employed, and of whom only three fell victims to this pestilence. Moreover, he affirms, that at no time is an unusual mortality to be observed among the factory-workers; on the contrary, he conceives that many diseases are avoided, through an absence of their causes, such as exposure to undue heat or cold, over-exertion, and the like causes, to which many other classes of artisans are exposed. Further, he says, it has been observed that the children who are engaged in factories, under careful supervision, are much less subjected to the ordinary causes of juvenile illness than those otherwise occupied.

No diseases are noticed to prevail endemically in those regions which are peopled by factory-hands; and the sickness and mortality are stated by our author to be in far greater proportion amongst the children of the villages unconnected with cotton-mills. The illness that actually does arise amongst the factory-labourers has its origin much oftener in the hours of recreation than in those of work; and when occurring it consists, for the most part, of some gastric derangement or catarrhal affection, which cannot well have had its cause in the apartments of labour. When the factory-children suffer and die from scrofulous disease, its germs are brought with them, and not contracted in the mills. Generally speaking, however, their state of health is most satisfactory, that of which every unprejudiced observer may convince himself by watching them at the periods of recreation, or on festival-days. In vain will a search be made for those unhappy and crippled youths who may have been discovered, probably, in some other states, and especially in England, the circumstances of which country differ so thoroughly from those of Austria, and which have given occasion to the philanthropic tirades (philanthropischen tiraden) of so many writers. Dr. Knolz winds up his remarks on this department of his inquiry by stating that it has been rendered quite certain, by a long experience, that, in thoracic disease, an abode in the warm atmosphere of a factory impregnated with oily particles, is very beneficial to all so affected; and thus, that children and old people often resist maladies of this kind years long, ever feeling themselves best whilst engaged in the workrooms.

We have now exhausted the substance of Dr. Knolz's pamphlet, so far as it relates to the influence of the factory system, in Lower Austria, upon the health and the general physical condition of the population, to whom it constitutes the source of subsistence. Although it is very likely that a little exaggeration in some of the details, and an unduly favorable colouring in the portraiture may occur, we yet feel no doubt that, in the main, our author is just and faithful. We think that the positions taken in our more extended article of last year, have here been strikingly corroborated, and their accuracy indeed demonstrated. We maintained, and again maintain, that working in cotton-mills is not *essentially* more prejudicial to health than the bulk of other kinds of labour, and that it is less so than many that have not occasioned the same degree of public sympathy and excitement. As an object of national polity, we are quite sure that it is its *regulation*, and not its *discouragement* that is needed; and, with respect to any legislative modification of the factory system in a sanitary point of view, we distinctly assert our conviction that further reduction in the hours of labour, involving, as it must, diminished wages as a consequence, will only, *as a beginning*, lead to an aggravation of the evils that beset the physical condition of the working classes; although, after every other practicable improvement shall have been accomplished, in the state alike of their homes and their work-places, such a proceeding may probably have many advantages, especially to the well-doing of the children. We take no cognizance of the economical or political considerations which attend this question; we have neither the knowledge nor the taste for the discussion of matters relating to the justice or expediency of interference, in special cases, with commercial enterprise, or of how far greater restrictions upon the duration of factory labour may have the effect of placing British capitalists at a disadvantage with foreigners; we have never studied these things. We feel confident, however, that, in all matters which affect the health and strength of the factory people of these kingdoms, we have some right to form a judgment; and this judgment before given we now reiterate, strengthened by perusal of the work we have partially analysed, that the ills which afflict this class of persons appertain to their *domestic* much more than to their *industrial* relations; and that the great mass of disease constantly witnessed in our manufacturing districts, flows from the *great town* rather than the *factory system*.

---

### Art. XIII.

*Mémoires de l'Académie Royale de Médecine.* Tome Dixième.—*Paris*, 1843. 4to, pp. 806.

*Memoirs of the Royal Academy of Medicine.* Vol. X.—*Paris*, 1843.

